# Anarchy in the UPR: a Ca^2+^-insensitive PKC inhibits SERCA activity to promote ER stress

**DOI:** 10.1042/BSR20170966

**Published:** 2018-03-16

**Authors:** Carsten Schmitz-Peiffer

**Affiliations:** 1 Diabetes and Metabolism Division, Garvan Institute of Medical Research, Sydney, NSW 2010, Australia; 2 St Vincent’s Clinical School, University of New South Wales, Sydney, NSW 2010, Australia

**Keywords:** ER stress, insulin resistance, lipogenesis, protein kinase C, steatosis

## Abstract

Nonalcoholic fatty liver disease (NAFLD) is highly prevalent in Western countries, and is linked to the development of liver cancer and Type 2 diabetes (T2D). It is strongly associated with obesity, but the dysregulation of liver lipid storage is not fully understood. Fatty acid oversupply to hepatocytes can establish a vicious cycle involving diminished protein folding, endoplasmic reticulum (ER) stress, insulin resistance and further lipogenesis. This commentary discusses the recent findings of Lai et al. published in *Bioscience Reports*, that implicate protein kinase C delta (PKCδ) activation by fatty acids in the inhibition of the SERCA Ca^2+^ pump, resulting in reduced ER Ca^2+^ loading and protein misfolding. PKCδ therefore represents a target for the treatment of both steatosis and insulin resistance, key to the prevention of NAFLD and T2D.

Nonalcoholic fatty liver disease (NAFLD), characterized by the accumulation of hepatic triglycerides and cholesterol, is now the most common chronic liver disease in Western countries, affecting up to 25% of the population [1]. The disease is associated with obesity and increases the risk of nonalcoholic steatohepatitis (NASH), fibrosis, cirrhosis, and hepatocellular carcinoma. In addition, NAFLD is linked to liver insulin resistance, a key feature of Type 2 diabetes (T2D) which is reaching epidemic proportions [2]. In combination with pancreatic β-cell dysfunction, insulin resistance leads to the failure of glucose homeostasis and results in long-term complications such as cardiovascular disease, kidney failure, and neuropathy. Therefore, the development of therapeutic strategies to prevent NAFLD are of key importance. However, liver lipid storage is normally tightly controlled in healthy subjects, and the mechanisms leading to its dysregulation are not well understood. In addition, the hierarchical relationship between excessive hepatic lipid deposition (steatosis) and insulin resistance is not clear, especially because there is evidence that each condition can play a causal role in the development of the other [3].

One mechanism that appears to link obesity and increased lipid availability to both steatosis and insulin resistance is the development of endoplasmic reticulum (ER) stress in hepatocytes [4]. If fatty acid delivery exceeds the ability of the hepatocyte to metabolize acyl chains by β-oxidation, the intracellular levels of triglyceride (TG) and the lipid intermediates in its synthesis will rise. Lipids have been proposed to negatively affect protein folding and trafficking in the ER in several ways [5–7], leading to the accumulation of unfolded proteins which in turn initiate the adaptive unfolded protein response, which increases protein folding capacity while reducing overall protein synthesis [8]. If this is insufficient to resolve the situation, chronic ER stress can lead to the induction of inflammatory pathways and insulin resistance, in part through Jnk activation and inhibition of insulin signal transduction [9].

Fatty acid oversupply can lead to perturbations in intracellular Ca^2+^ homeostasis which in turn diminish protein folding capacity by affecting chaperone activity [10]. Lipid accumulation in hepatocytes has been reported to affect Ca^2+^ entry into cells through store-operated Ca^2+^ channels [11] and also to decrease both the activity [6] or expression [12] of the sarco/endoplasmic reticulum calcium ATPase (SERCA), a key component of Ca^2+^ homeostasis that transports Ca^2+^ ions into the ER. However, the mechanism by which lipids affect SERCA has not been elucidated.

Recently, in *Bioscience Reports*, Lai et al. show [13] that protein kinase C delta (PKCδ), a member of the lipid-activated PKC family, regulates SERCA function in LO2 hepatocytes treated with the fatty acid palmitate, an *in vitro* model of hepatic steatosis. The PKC family of kinases consists of 10 isoforms grouped by their sensitivity to activators. The conventional PKCs (PKCα, PKCβI, PKCβII and PKCγ) are Ca^2+^ dependent, whereas the novel PKCs (PKCδ, PKCε, PKCθ, and PKCη) and atypical PKCs (PKCζ and PKCι/λ) are not. Both conventional and novel PKCs are, however, sensitive to diacylglycerol (DAG). This lipid is a second messenger released predominantly at the plasma membrane by acute phospholipase C (PLC) activation in response to extracellular signals, but is also an intermediate in fatty acid metabolism that accumulates at the ER in cells and tissues exposed to lipid excess [14]. Through the phosphorylation of their protein substrates, PKC isoforms regulate a wide variety of cellular processes, including gene expression, cell growth and division, and cytoskeletal reorganization [15]. The conventional and especially the novel isoforms are also important candidates for mediating the effects of lipid oversupply, because of their activation by DAG [14].

When cells sequester fatty acids as TG in lipid droplets, the flux through the DAG intermediate occurs as a result of the actions of acyl transferases, that esterify fatty acids onto a glycerol phosphate backbone, and lipins (phosphatidic acid phosphatases) that subsequently remove the phosphate group to generate DAG [16]. Finally, diacylglycerol acyltransferases esterify further fatty acid molecules with DAG to form TG which enters lipid droplets forming at the ER. DAG generated in this way is therefore well-situated to activate PKCδ at a location where SERCAs are found, in contrast with second messenger DAG molecules released from plasma membrane phospholipids by PLC. In each case, the DAG stereoisomer generated (sn-1,2 DAG) is capable of activating PKC [17], whereas the DAG stereoisomers released from lipid droplets during lipolysis (primarily sn-1,3 and sn-2,3 DAG) are unlikely to participate [18].

Lai et al. demonstrate that palmitate-treated LO2 cells accumulate TG in lipid droplets, and this is associated with not only an increase in the expression of ER stress markers and dysregulation of intracellular Ca^2+^, but also the activation of PKCδ [13]. Furthermore, knockdown of PKCδ improves intracellular Ca^2+^ regulation and reduces the induction of ER stress markers by the lipid, as well as reducing TG accumulation itself. A key finding is the restoration of SERCA activity, which is greatly reduced by palmitate treatment, through PKCδ depletion. This occurs in the absence of an effect on SERCA2 protein levels, suggesting a modulation of ATPase function rather than expression.

This is a novel observation which further implicates PKCδ as a mediator of the deleterious effects of lipid excess, and as a potential therapeutic target. PKC activation has previously been implicated in the reduction of ER Ca^2+^ content [19], and the effects of PKCδ on SERCA function may be mediated directly through phosphorylation of the protein. This would be similar to the PKC-dependent phosphorylation and inhibition of store-operated Ca^2+^ channels [11,20], and such a mechanism would be fully consistent with the alleviation of ER stress by PKCδ knockdown.

On the other hand, the data reported by Lai et al. lend support to the idea that PKCδ represents a key node in a more complex network of interactions between lipid oversupply, ER stress and insulin resistance. While it has long been proposed that aberrant PKC activity directly interferes with proximal insulin signaling and hence with the suppression of hepatic glucose output [21], investigations of PKC isoform deletion in mice have also implicated other roles, including the induction of ER stress [22,23] and the dysregulation of fatty acid metabolism itself [24]. Indeed, it appears that PKC isoforms not only respond to intracellular lipid signals, but also in turn regulate *de novo* lipid synthesis, fatty acid esterification, lipolysis, and β-oxidation [14]. Thus PKCδ deletion reduces lipogenesis in the liver of mice fed a high fat diet [25], which may be mediated by increased signaling to the lipogenic transcription factor Srebp1c through an mTORC1/p70S6K-dependent manner [26]. This would represent a positive feed-back loop, since further fatty acid synthesis promoted by PKCδ is likely to lead to additional elevations in intracellular DAG. Deletion of PKCδ in mice also promotes hepatic insulin sensitivity and improved glucose homeostasis [26], which could be due direct effects at the level of proximal insulin signaling, or indirectly through alleviation of lipid accumulation or ER stress. Supporting the latter, Greene et al. showed that PKCδ levels and activation were increased in *in vivo* and cellular models of NASH, induced by methionine and choline deficiency (MCD), together with markers of ER stress [27]. PKCδ knockdown alleviated ER stress, although the link between PKCδ and ER stress also appeared to be bidirectional, in that ER stress also contributed to PKCδ activation [27], as also reported for TNFα-induced ER stress [28].

Consistent with the *in vivo* studies of high fat diet-fed PKC-deficient mice [23,25,26], but differing from the findings of MCD [27], Lai et al. also observed that knockdown of PKCδ reduced the accumulation of TG and lipid droplets in palmitate-treated LO2 hepatocytes [13]. This supports the idea of a more complex interaction between PKCδ, fatty acid excess, ER stress, and steatosis. If a simple, linear hierarchy existed from fatty acids, through increased DAG flux at the ER, to PKCδ activation and Ca^2+^ dysregulation, then PKCδ knockdown would not be expected to reduce lipid accumulation, even though ER stress would be alleviated by restoration of SERCA activity. However, alterations in Ca^2+^ homeostasis have been linked to subsequent TG formation [29], and a vicious cycle appears to exist, in which the ER stress resulting from fatty acid oversupply leads to further lipid accumulation, due either to increased lipogenesis or to reduced β-oxidation [30]. While Ca^2+^ overloading of mitochondria through increased contact with the ER can reduce β-oxidation [31], this mechanism is unlikely to pertain when SERCA activity is reduced and ER Ca^2+^ loading is therefore diminished. Instead, ER stress may have induced the expression of lipogenic machinery such as fatty acid synthase through activation of Srebp1c [6]. This transcription factor is located at the ER and can be activated upon ER stress [30], and also appears to be another downstream target of PKCδ [26]. In overall support of such a cycle, treatment of *ob/ob* obese mice with an allosteric activator of Serca2b ameliorates hepatic steatosis, with down-regulation of lipogenic genes [32]. A summary of these interconnected mechanisms is presented in Figure 1.

**Figure 1:**
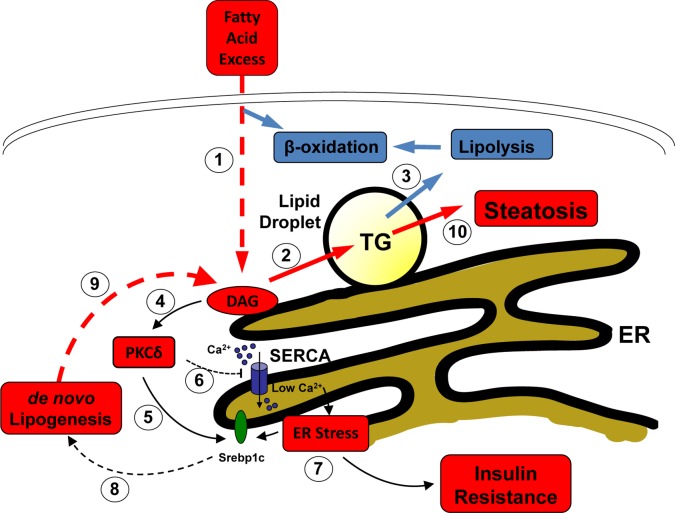
Interactions that link fatty acid oversupply to steatosis and insulin resistance through PKCδ activation and ER stress When hepatocytes are exposed to fatty acid oversupply, intracellular levels may exceed the amount that can be metabolized by direct β-oxidation, leading to increased esterification (**1**) and flux through diacylglycerol (DAG) intermediates to sequestration in lipid droplets (**2**) as triglyceride (TG). In healthy cells this will be released by lipolysis for subsequent β-oxidation (**3**). However, excessive DAG accumulation causes chronic activation of PKCδ (**4**) which can promote signaling to the lipogenic transcription factor Srebp1c (**5**). In addition, Lai et al. now show that PKCδ activation leads to inhibition of SERCA activity and a reduction in ER Ca^2+^ (**6**). This in turn promotes ER stress which can also directly activate Srebp1c as well as induce insulin resistance (**7**). Enhanced lipogenesis (**8**) gives rise to further DAG levels (**9**) establishing a vicious cycle which overwhelms the ability of the hepatocyte to deal with TG accumulation and results in steatosis (**10**). See the text for further details.

In contrast, while another novel PKC isoform, PKCε, is also strongly linked to liver insulin resistance, ablation of this kinase in mice is associated with mildly increased hepatic TG synthesis after a week of fat feeding [24], suggesting a negative feedback pathway dependent on the enzyme. This appears to dissociate lipid accumulation and PKCε activation from the generation of liver insulin resistance, and similar discrepancies regarding hepatic DAG levels and insulin action have been reported [33–35]. Alternatively, it suggests the existence of distinct pools of DAG which do not all affect insulin sensitivity [34]. An alteration in lipid partitioning towards esterification rather than β-oxidation may also protect hepatocytes during acute fatty acid excess, avoiding the accumulation of reactive oxygen species. In any case, it is clear that PKCε and PKCδ, while both potential targets for the treatment of insulin resistance and glucose intolerance, play non-redundant roles in lipid metabolism.

A PKCδ-dependent stimulation of lipogenesis through ER stress may also account in part for the selective insulin resistance of glucose metabolism but not of lipogenesis that can occur in hepatocytes [36]. While unresolved ER stress can lead to Jnk activation and interference with proximal insulin signaling, so that the down-regulation of gluconeogenic genes through the Akt2-FoxO1 pathway is diminished, the concomitant activation of Srebp1c through ER stress may augment the effects of the hormone on lipid synthesis [30]. DAG-activated signaling through PKCδ would be expected to promote this paradoxical state.

A question that is raised by the work of Lai et al. is the nature of the effect of PKCδ on SERCA activity. The Ca^2+^-sensitive conventional isoform PKCα inhibits SERCA2 activity in cardiomyocytes by directly phosphorylating protein phosphatase inhibitor-1. This in turn promotes the dephosphorylation of the SERCA2 inhibitor phospholamban and its interaction with SERCA2, diminishing contractility by reducing sarcoplasmic reticulum Ca^2+^ loading and release [37]. Whether PKCδ plays a role in a similar mechanism, or even directly phosphorylates SERCA, is not known. Interestingly, a key difference is the Ca^2+^-insensitivity of this novel PKC. While PKCα can mediate Ca^2+^-dependent feedback inhibition, PKCδ provides a nexus between lipid signaling and Ca^2+^ homeostasis.

The report by Lai et al. thus provides a new insight into the role of PKC in the development of steatosis and insulin resistance. An effect of PKCδ on SERCA activity and thus Ca^2+^ homeostasis and ER stress may help to explain some of the links between these pathologies, and further highlights the potential therapeutic value in targeting this family of kinases for the treatment of obesity-related disease.

## Abbreviations

DAG: diacylglycerol
ER: endoplasmic reticulum
MCD: methionine and choline deficiency
NAFLD: nonalcoholic fatty liver disease
NASH: nonalcoholic steatohepatitis
PKCδ: protein kinase C delta
PLC: phospholipase C
TG: triglyceride
T2D: Type 2 diabetes
